# Use of probiotics in the treatment of severe acute pancreatitis: a systematic review and meta-analysis of randomized controlled trials

**DOI:** 10.1186/cc13809

**Published:** 2014-03-31

**Authors:** Shanmiao Gou, Zhiyong Yang, Tao Liu, Heshui Wu, Chunyou Wang

**Affiliations:** 1 Department of General Surgery, Pancreatic Disease Institute, Union Hospital, Tongji Medical College, Huazhong University of Science and Technology, 1277 Jiefang Avenue, Wuhan, Province Hubei 430022, China

## Abstract

**Introduction:**

Necrotic tissue infection can worsen the prognosis of severe acute pancreatitis (SAP), and probiotics have been shown to be beneficial in reducing the infection rate in animal experiments and primary clinical trials. However, the results of multicenter randomized clinical trials have been contradictory. Our aim in this study was to systematically review and quantitatively analyze all randomized controlled trials with regard to important outcomes in patients with predicted SAP who received probiotics.

**Methods:**

A systematic literature search of the PubMed, Embase and Cochrane Library databases was conducted using specific search terms. Eligible studies were randomized controlled trials that compared the effects of probiotic with placebo treatment in patients with predicted SAP. Mean difference (MD), risk ratio (RR) and 95% confidence interval (95% CI) were calculated using the Mantel-Haenszel fixed- and random-effects models. A meta-analysis on the use of probiotics in the treatment of critically ill patients was also performed to serve as a reference.

**Results:**

In this study, 6 trials comprising an aggregate total of 536 patients were analyzed. Significant heterogeneities were observed in the type, dose, treatment duration and clinical effects of probiotics in these trials. Systematic analysis showed that probiotics did not significantly affect the pancreatic infection rate (RR = 1.19, 95% CI = 0.74 to 1.93; *P* = 0.47), total infections (RR = 1.09, 95% CI = 0.80 to 1.48; *P* = 0.57), operation rate (RR = 1.42, 95% CI = 0.43 to 3.47; *P* = 0.71), length of hospital stay (MD = 2.45, 95% CI = −2.71 to 7.60; *P* = 0.35) or mortality (RR = 0.72, 95% CI = 0.42 to 1.45; *P* = 0.25).

**Conclusions:**

Probiotics showed neither beneficial nor adverse effects on the clinical outcomes of patients with predicted SAP. However, significant heterogeneity was noted between the trials reviewed with regard to the type, dose and treatment duration of probiotics, which may have contributed to the heterogeneity of the clinical outcomes. The current data are not sufficient to draw a conclusion regarding the effects of probiotics on patients with predicted SAP. Carefully designed clinical trials are needed to validate the effects of particular probiotics given at specific dosages and for specific treatment durations.

## Introduction

Acute pancreatitis (AP) is a common disease that affects about 270,000 people annually in the United States, and its incidence has been increasing by 5% every year in the United States and Europe [1,2]. Severe acute pancreatitis (SAP) accounts for 10% to 20% of AP cases and has unacceptably high morbidity and mortality rates [3,4]. Necrotic tissue infection is one of the principal causes of complications and death in SAP patients. It is believed that intestinal barrier dysfunction and subsequent bacterial translocation from the intestinal tract to the bloodstream and necrotic tissues play a critical role in the infection of necrotic tissues [5-7]. Evidence derived from animal studies suggests that probiotics could stabilize the intestinal barrier and thus minimize bacterial translocation and prevent infection in AP [8-11]. Moreover, clinical trials have documented the benefits of probiotics in some critically ill patients [12-18]. The results of several studies indicate that probiotics can enhance intestinal barrier function, stimulate host cell production of antimicrobial peptides and produce antimicrobial factors, so probiotics could potentially be effective in the treatment of SAP [19-22]. Clinical trials have been conducted on the use of probiotics in AP patients at risk of developing SAP. However, disparate results were obtained from the trials; therefore, there is still no consensus about the use of probiotics in SAP [23-28], and their use is rarely recommended in clinical practice guidelines [2,29-38].

The results of the PROPATRIA trial (probiotic prophylaxis in patients with predicted severe acute pancreatitis), published in 2008 (a multicenter randomized controlled trial (RCT) dominated by the Dutch Acute Pancreatitis Study Group), showed that probiotics had harmful effects [23], which deterred the initiation of other trials on probiotics. In recent years, however, two other RCTs have been completed, with no negative consequences were in patients treated with probiotics [24,28]. We therefore consider that the results of the PROPATRIA trial are questionable and that further meta-analyses of the more recent RCTs is required. To this end, we performed this meta-analysis on six select RCTs in order to determine the effects of probiotics on the rate of pancreatic and total infection, operation rate, length of hospital stay and mortality. In addition, we tried to determine the reason for the heterogeneous results across the different trials.

## Methods

### Systematic literature search

A systematic literature search was performed in the PubMed, Embase and Cochrane Library databases. The search was restricted to human evidence published since 1992, which was the year in which the term *severe acute pancreatitis* was approved at the Atlanta symposium. The MeSH headings *pancreatitis*, *Lactobacillus*, *prebiotics*, *synbiotics* and *probiotics* were used, but the language was not restricted to English only. Articles were compiled into a database, and duplicates were removed. The abstracts were then screened for relevance. In the case of multiple articles published by the same study group for the same study period, only the most recent paper was selected. Subsequently, full-text papers of the selected studies published in English were screened for eligibility. If the study was published in another language, we contacted the corresponding author to check whether it had been translated into English. The translated studies were included, and nontranslated studies were eliminated. A systematic literature search for probiotic use in critical illness was also performed. An additional Microsoft Word file describing this search in more detail is available in Additional file 1. No participant consent were needed for this review, as it evaluated published studies without individually identifiable patient information.

### Inclusion and exclusion criteria

We included all human RCTs in which the effects of probiotics in SAP patients were investigated. We excluded (1) trials that included patient cohorts with mild AP and in which the SAP results were not reported separately and (2) studies that included cohorts for which the essential outcomes were not reported. The search and inclusion or exclusion of articles were carried out by two authors (SG and TL). In cases of uncertainty or disagreement, a third author was consulted (CW).

### Data extraction

From among the included studies, the following variables were extracted (if available): definition of predicted SAP, sample size, types of probiotics used and duration of use, intervention in the control group and clinical outcomes. The internal validity was determined using the Jadad score [39] and six quality criteria of the Cochrane Collaboration: random sequence generation, allocation concealment, blinding of participants and personnel, blinding of outcome assessment, incomplete outcome data and selective data reporting. Any publication bias was not evaluated.

### Statistical analysis

All pooled data were analyzed using Review Manager (RevMan) version 5.2.6 software (Cochrane Informatics and Knowledge Management Department). Risk ratio (RR) with 95% confidence interval (CI) was used for dichotomous outcomes, and mean difference (MD) was used for continuous outcomes. The χ^2^ test was used to assess heterogeneity between trials, and *I*^2^ values were used to assess the extent of inconsistency. The Mantel-Haenszel random-effects model and fixed-effects model were used to analyze data with and without significant heterogeneity, respectively. Two-sided *P*-values <0.05 were considered to indicate statistical significance.

## Results

### Included studies

**Figure 1:**
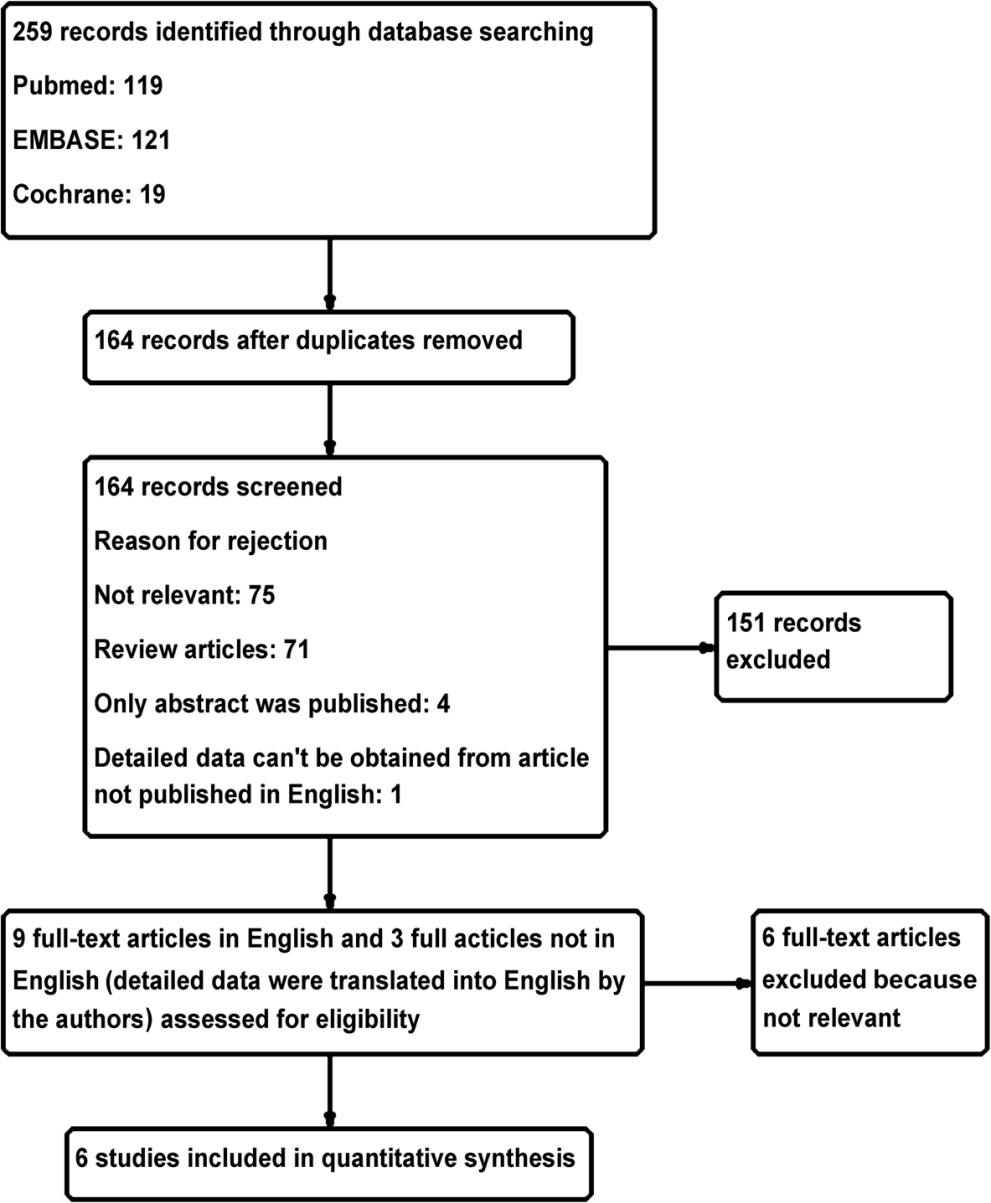
Flowchart illustrating the details of the search and study selection process.

The results of the literature search are depicted in Figure 1. An initial search of the databases yielded a total of 259 articles. After screening titles and abstracts for relevance, 12 articles were assessed further for eligibility. Of the twelve articles, six were excluded: one was a retrospective study, three articles reported results of the same study, one article did not report the outcomes of mixed cohorts with severe and mild acute pancreatitis separately and, in one article, the experimental patients received probiotic-containing enteral nutrition (EN) and control patients received parenteral nutrition (PN) but not probiotic-free EN. Thus, six RCTs were ultimately included in our present systematic review [23-28]. The blinding, randomization, random sequence generation, withdrawals and dropouts, and allocation concealment for all the trials are listed in Table 1. The Jadad scores of the six trials ranged from 1 to 5. On the basis of their Jadad scores, four of the trials were of low quality (≤2) and two were of high quality (>2). An additional Microsoft Word file showing the risks of bias in more detail is in Additional file 2. Most of the trials had a high or unclear risk of selection bias, performance bias, detection bias and reporting bias. Each outcome reported in our present study is based on the results of only three to five trials; therefore, publication bias was not evaluated [40].

**Table 1:** Information about the six trials

**Study**	**Year**	**Country**	**Blinding**	**Randomization**	**Random sequence generation**	**Withdrawals and dropouts**	**Allocation concealment**	**Jadad score**
Oláh *et al*. [26]	2002	Hungary	Unclear	Yes	Unclear	0/0	Unclear	2
Li *et al*. [25]	2007	China	None	Yes	Unclear	Unclear	Unclear	1
Oláh *et al*. [27]	2007	Hungary	Double-blinded	Yes	Unclear	0/0	Yes	3
Besselink *et al*. [23]	2008	Netherlands	Double-blinded	Yes	Computer-generated, permuted-block sequence	2/5	Yes	5
Plaudis *et al*. [28]	2012	Latvia	Unclear	Unclear	Unclear	0/0	Unclear	1
Cui *et al*. [24]	2013	China	None	Yes	Unclear	Unclear	Unclear	1

### Types of probiotics, doses and treatment duration

In the six RCTs included in the meta-analysis, a total of 14 strains of probiotic bacteria were used (Table 2). One trial used a single strain, and the other five used a combination of probiotics containing three to six strains. Only two trials used the same mixture of probiotics: *Pediococcus pentosaceus*, *Leuconostoc mesenteroides*, *L. paracasei* and *L. plantarum* (Synbiotic 2000 Forte; Medifarm, Kågeröd, Sweden). In total, *L. plantarum* was used in three trials: *Bifidobacterium longum*, *L. bulgaricus*, *L. paracasei*, *L. mesenteroides* and *P. pentosaceus* were used in different combinations in two trials, and *B. bifidum*, B. lactis, *Enterococcus* faecalis, L. acidophilus, L. casei, L. lactis, L. salivarius and *Streptococcus. thermophilus* were used in just one trial. The lowest daily dose of probiotics was 3 × 10^7^ bacteria, and the highest dose was 8 × 10^8^ bacteria. The length of treatment was reported clearly in five studies, ranging from 7 to 28 days and not reported clearly in one study [28]. In three trials, the duration of treatment was 7 days [25-27], and the duration was 14 days in one study [24] and 28 days in one trial [23].

**Table 2:** Types of probiotics used in the trials

**Trial**	**Probiotics group**	**Control group**	**Length of treatment (days)**
Oláh *et al*. [26]	10^9^*Leuconostoc plantarum* 299 per serving	10^9^ heat-inactivated *L. plantarum* 299 per serving	7
	Twice daily	Twice daily	
Li *et al*. [25]	10^7^*Bifidobacterium longum*, 10^6^*L. bulgaricus*, and 10^6^*Streptococcus thermophilus* per serving (Golden Bifid)	Water	7
	Thrice daily	Three times daily	
Oláh *et al*. [27]	10^10^*Pediococcus pentosaceus*, 10^10^*L. mesenteroides*, 10^10^*L. paracasei* and 10^10^*L. plantarum* with bioactive fibers per serving (Synbiotic 2000 Forte; Medifarm, Kågeröd, Sweden)	Bioactive fibers	7
	Once daily	Once daily	
Besselink *et al*. [23]	*L. acidophilus*, *L. casei*, *L. salivarius*, *L. lactis*, *B. bifidum* and *B. lactis* in a totally daily dose of 10^10^ (Ecologic 641; Winclove Bio Industries, Amsterdam, the Netherlands)	Placebo	28
	Twice daily	Twice daily	
Plaudis *et al*. [28]	10^10^*P. pentosaceus*, 10^10^*L. mesenteroides*, 10^10^*L. paracasei* and 10^10^*L. plantarum* with bioactive fibers per serving (Synbiotic 2000 Forte)	Bioactive fibers	Unclear
	Twice daily	Twice daily	
Cui *et al*. [24]	4 × 10^7^*B. longum*, 4 × 10^7^*L. bulgaricus* and 4 × 10^7^*Enterococcus faecalis* per serving	Water	14
	Twice daily	Twice daily	

### Other baseline characteristics

In the aggregate, the six included studies comprised 536 participants, and the number of participants per study ranged from 25 to 298. Among the 536 participants, 275 received EN with probiotics and 261 received probiotic-free EN. The prediction variables for SAP varied between the trials: an Acute Physiology and Chronic Health Evaluation II (APACHE II) score ≥8 was considered as an indicator in three studies [23-25], Glasgow (Imrie) score ≥3 in four studies [23,24,26,27], C-reactive protein (CRP) level ≥150 mg/L in three studies [23,26,27] and extensive pancreatic necrosis in two studies [24,25]. In one study, SAP was predicted by APACHE II score ≥6 with systemic inflammatory response syndrome and/or organ dysfunction [28].

The relevant baseline characteristics were reported in detail in three studies [23,26,27], but were limited in the other three studies [24,25,28]. Overall, no significant differences in the baseline characteristics between the probiotics and control groups were reported. Only Besselink *et al*. [23] reported multiple organ failure (MOF), which occurred in 2% of the patients before randomization. The incidence of MOF before randomization, which plays a key role in the mortality associated with SAP, was 3.3% and 0.7% in the probiotics group and control groups, respectively (*P* = 0.24) [23]. Researchers in three of the studies reported a male:female ratio of 1.80 and a mean age of 56 years [23,26,27]. In three studies, the investigators reported that the proportion of patients with alcoholic pancreatitis was 30.6% [23,26,27]. The Glasgow score was reported in three studies, and the APACHE II score was reported in two studies. The mean Glasgow score was 3.2 [23,26,27], and the mean APACHE II score was 8.54 [23,28]. The plasma CRP level was reported in four studies, with an overall mean of 264.5 mg/L [23,24,26,27]. In four studies, the proportion of patients with necrotizing pancreatitis was 37% [23,26-28].

### Clinical outcomes

**Figure 2:**
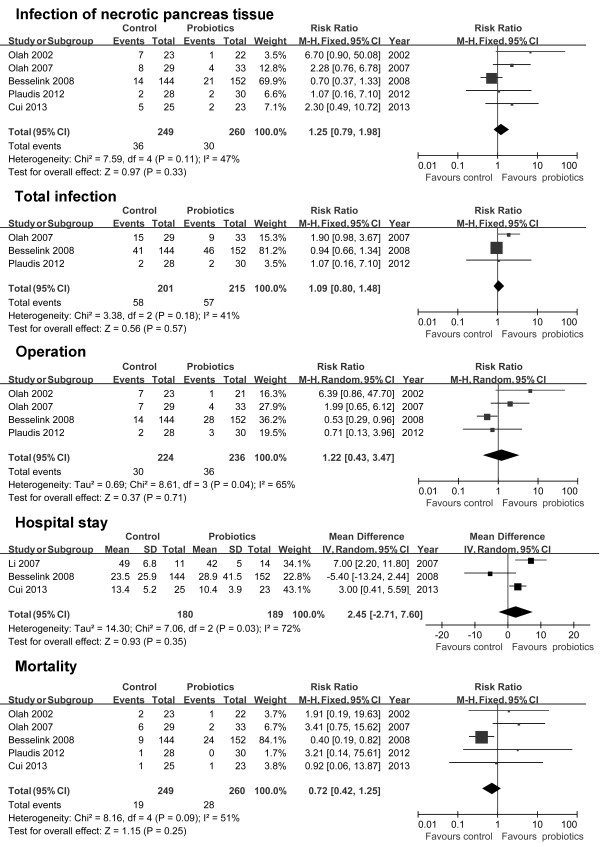
**Tabulated data and forest plots illustrating the effects of probiotics on the clinical outcomes of patients with predicted severe acute pancreatitis.** CI, Confidence interval; IV, Inverse variance; M-H, Mantel-Haenszel.

The clinical outcomes are shown in Figure 2. In five studies comprising an aggregate total of 509 patients, the researchers reported the incidence of pancreatic infection. The difference between the probiotics and control groups was not significant in any of the five studies (RR = 1.25, 95% CI = 0.79 to 1.98; *P* = 0.33). Moreover, there was no significant heterogeneity between the studies (*I*^2^ = 43%; *P* = 0.11).

Researchers in three studies reported total infection rates in an aggregate of 416 patients. A significantly lower total infection rate was observed in the probiotics group compared to the control group in one study (Oláh *et al*. [27]), and the difference between the groups was not significant in the other two studies. Overall, no significant difference was observed between the probiotics group and control group (RR = 1.09, 95% CI = 0.80 to 1.48; *P* = 0.57). Moreover, there was no significant heterogeneity between the studies (*I*^2^ = 41%; *P* = 0.18).

In four studies comprising a total of 460 patients, researchers reported the number of patients who underwent surgery. In one study, a significantly higher number of patients in the probiotics group than in the control group underwent surgery (Besselink *et al*. [23]). The difference between the groups was not significant in the other three studies. Overall, no significant difference was observed between the probiotics group and the control group (RR = 1.42, 95% CI 0.43 to 3.47; *P* = 0.71). The heterogeneity between studies was significant (*I*^2^ = 65%; *P* = 0.04).

In three studies comprising 369 patients, the investigators reported the duration of hospital stay. Significantly shorter hospital stays were reported in the probiotics group in two studies (Li *et al*. [25] and Cui *et al*. [24]), and no significant difference was observed in the other study. Overall, no significant difference in length of hospital stay was observed between the probiotics group and the control group (MD = 2.45, 95% CI = −2.71 to 7.60; *P* = 0.35). The heterogeneity between studies was significant (*I*^2^ = 72%; *P* = 0.03).

In five studies comprising 509 patients, researchers reported the mortality rates. Significantly higher mortality was observed in the probiotics group in one study (Besselink *et al*. [23]), and no significant difference between the probiotics and control groups was observed in the other four studies. Overall, no significant difference in length of hospital stay was observed between the probiotics and control groups (RR = 0.72, 95% CI = 0.42 to 1.45; *P* = 0.25). There was no significant heterogeneity across studies (*I*^2^ = 51%; *P* = 0.09).

We analyzed subgroups by treatment duration. Additional file 3 gives the the results in more detail. In the subgroup in which the treatment duration was within 15 days or less, a significant decrease in pancreatic infection was observed in the probiotics group compared to the control group (RR = 2.94, 95% CI = 1.31 to 6.60; *P* < 0.01). We also performed a meta-analysis of the use of probiotics in patients with critical illness with subgroup analyses conducted according to the antibiotic types and treatment durations. Additional files 4 and 5 show the results of these analyses in detail. We observed significant differences between patients taking different types of probiotics or probiotic mixtures (see Additional file 4). The subgroup in which the treatment duration was within 15 days or less showed better treatment effects than the subgroup in which the treatment duration was not limited to 15 days with regard to total infections (RR = 1.49, 95% CI = 1.15 to 1.93, *P* < 0.01 vs. RR = 1.19, 95% CI = 0.98 to 1.45, *P* = 0.09) and pneumonia (RR = 1.81, 95% CI = 1.27 to 2.59, *P* < 0.01 vs. RR = 1.41, 95% CI = 1.09 to 1.84, *P* = 0.01) (see Additional file 5).

## Discussion

In this meta-analysis of RCTs that compared probiotics with placebo in patients with SAP, we found no evidence for probiotics’ being either harmful or beneficial with regard to all important outcomes. However, significant heterogeneity was found between different trials, so the results need to be validated further.

Following the harmful effects of probiotics in SAP reported in the PROPATRIA trial [23], Sun *et al*. reported a meta-analysis of four RCTs with significant heterogeneity [41]. In recent years, two other RCTs have been conducted on the same subject; therefore, we conducted our present meta-analysis of all six trials with a focus on the source of the heterogeneity. The results indicate that the type and duration of treatment may contribute greatly to the heterogeneity of the results. The results of the subgroup analysis would be underpowered, with only six RCTs included in the meta-analysis. To compensate for this, we also analyzed the anti-infective effect of probiotics on the treatment of critical illness to serve as a reference.

The performance of strains differs, as different bacteria have different adherence sites and divergent immunological effects [42,43]. Therefore, the types of probiotics used may have led to differences in the clinical outcomes. *L. plantarum* 299, Synbiotic 2000 Forte and golden bifid, which were used in RCTs for the treatment of predicted SAP [25-28] have also been used in RCTs for the treatment of critical illness [13,14,17,44-50]. The probiotic mixture used in the Cui *et al*. trial contained probiotic strains similar to golden bifid [24]. These probiotics resulted in significant improvement of infection or showed a trend in that direction. However, we found no RCTs on critical illness in PubMed in which Ecologic 641 (Winclove Bio Industries, Amsterdam, the Netherlands) was used for treatment (Besselink *et al*. [23], PROPATRIA trial), and none of the six strains of probiotic bacteria in Ecologic 641 were used in the other five trials studied in the present meta-analysis. Further, probiotic mixtures that contained probiotic strains similar to Ecologic 641 were studied in only two of the thirteen RCTs on critical illness (Jain *et al*. [51] and Barraud *et al*. [52]), and both of these RCTs showed that probiotics had a detrimental effect on infection, although the effect was not significant (see Table 2, Figure 2 and Additional file 4). All these findings suggest that the type of probiotic used plays an important role in the heterogeneity between trials.

The second aspect to be considered is the dose of probiotics, as the same probiotics can have opposite effects at different doses [53]. The dose of probiotics varied greatly among the six RCTs in this meta-analysis. Even when the same probiotics were used in different trials, the doses were different. Thus, the dose may have also contributed to the heterogeneity.

The final factor to be considered is the duration of treatment, although it is still not clear whether the effects of probiotics are influenced by this variable [54]. An interesting finding was that the subgroup in which the treatment duration was within 15 days or less showed significant improvement with regard to almost all outcomes in the probiotics groups (see Additional file 3). Similarly, trials for the treatment of critical illness with treatment duration of no more than 15 days also showed higher efficacy with regard to decreased infection and reduced heterogeneity (see Additional file 5). We tentatively put forth the notion that prolonged treatment duration may lead to an overload of probiotics, which might be harmful to patients with SAP and critical illness who have intestinal barrier dysfunction. However, there is a need for evidence to confirm this hypothesis, as the results could be artefactually positive.

The PROPATRIA trial, which was the best in terms of methodological quality (Jadad score of 5), also had the highest number of participants, and the investigators used Ecologic 641 for the treatment of patients with predicted SAP, but with no beneficial effects. Some ongoing studies were abandoned after the dismal results of the PROPATRIA trial [55], and even the executors of the PROPATRIA trial thought that new randomized trials of probiotics in patients with predicted SAP were not warranted [56]. However, the dose, type and treatment duration, which have been documented to be critical factors, vary in clinical trials. Ecologic 641, which has rarely been used in other clinical trials, induced high concentrations of interleukin 10 (IL-10) and low concentrations of IL-2 compared with the other probiotics. This effect is considered to have contributed to bowel ischemia in the probiotic-treated patients in the PROPATRIA trial [57]. Therefore, the results of the PROPATRIA trial are not sufficient to draw the conclusion that probiotics are associated with adverse effects in the treatment of patients with predicted SAP.

The history of probiotic treatment of SAP is similar to that of immune formula treatment of critical illnesses. Both treatments were documented to be beneficial in animal experiments and primary clinical trials, but subsequent multicenter, prospective, randomized clinical trials showed adverse effects, which made the efficacy of the treatments questionable [58]. However, subsequent studies of immune formulations indicated that certain populations of patients did benefit from the treatment [59,60]. Similarly, because the trials were heterogeneous with regard to their treatment strategies and outcomes, it is still possible that patients with SAP can benefit from particular probiotics if administered at the appropriate dose and for the correct duration. Moreover, given that prophylactic antibiotics have been tried with limited or no success, the effects of probiotics with regard to their potential in decreasing necrotic tissue infection warrant further study.

In the interpretation of the results of this meta-analysis, the limited number of available trials, the methodological quality of the included trials and the heterogeneity of the included trials should be noted. Only six RCTs with relatively low methodological quality were included (that is, Jadad score <3 for four of the six included trials), and considerable heterogeneity was observed among these trials with regard to the effect of probiotics. The limited number of RCTs hampered the subgroup analysis conducted to understand the cause of the heterogeneity. Upon reviewing 11 major clinical practice guidelines for AP [2,29-38], we found that the use of probiotics was mentioned only in the Society of Critical Care Medicine [32] and International Association of Pancreatology and American Pancreatic Association guidelines [29]. Therefore, because of insufficient clinical data, there is clearly a need for further investigations.

## Conclusions

Although the results indicate that probiotics had neither beneficial nor adverse effects, the current data are not sufficient to draw conclusions about the effects of probiotics in patients with SAP, so further studies are needed. However, the types of probiotics and treatment strategies used vary considerably across studies, so probiotic/probiotic mixtures, their appropriate dosages and treatment duration must be considered carefully before clinical trials are conducted. Moreover, considering the risks reported for certain probiotics, further clinical trials should be carefully designed to avoid any potentially harmful effects. In addition, RCTs on patients with critical illness can serve as a reference for future studies on SAP treatment.

## Key messages

● The current data are not sufficient to draw conclusions about the effects of probiotics in patients with SAP because of the limited number of trials and their heterogeneity. Nonetheless, the possibility cannot be ruled out that patients with predicted SAP can benefit from particular probiotics when administered at the appropriate dose and for the correct duration.

● The types of probiotics used and the treatment strategies employed play an important role in the heterogeneity of clinical outcomes reported in different RCTs.

● Further investigations on the effects of probiotics in patients with SAP are needed, but further clinical trials should be carefully designed to avoid any potentially harmful effects. RCTs in patients with critical illness can serve as a reference.

## Abbreviations

AP: Acute pancreatitis; CI: Confidence interval; CRP: C-reactive protein; EN: Enteral nutrition; MD: Mean difference; MOF: Multiple organ failure; RCT: Randomized controlled trial; RR: Risk ratio; SAP: Severe acute pancreatitis.

## Competing interests

The authors declare that they have no competing interests.

## Authors’ contribution

SG, ZY, TL, HW and CW were involved with study conception and design. SG, ZY and TL were involved in data acquisition. SG, ZY, HW and CW interpreted the data and results of the analyses. SG and ZY drafted the manuscript, which was critically revised for intellectual content by TL, HW and CW. All authors read and approved the final manuscript.

## Authors’ information

SG and TL are pancreatic disease specialists. SG is also an intensivist at the Pancreatic Disease Institute (PDI), Union Hospital of Huazhong University of Science and Technology (HUST). ZY is a pancreatic disease specialist and the resident of the Clinician Investigator Program who is currently performing a study about fluid resuscitation in severe acute pancreatitis. HW is the deputy head of the PDI, Union Hospital of HUST. CW is the head of the PDI, the head of the Department of Surgery and the Director of the Surgical Teaching and Research Section of Union Hospital of HUST. CW is also the Vice Chairman of the Pancreatic Disease Group of the Chinese Medical Association, the Vice Chairman of the Pancreatic Cancer Committee of the Chinese Anti-Cancer Association and the Chairman of the Hubei Pancreatic Disease Association.

## Supplementary Material

Additional file 1 Systematic literature search for studies on the use of probiotics in critical illness. Click here for file

Additional file 2 Risk of bias. Click here for file

Additional file 3 Analysis of subgroups by treatment duration in predicted SAP. Click here for file

Additional file 4 Analysis of subgroups by types of probiotics used in critical illness. Click here for file

Additional file 5 Analysis of subgroups by treatment duration in critical illness. Click here for file
