# Supplementary material for: Use of probiotics in the treatment of severe acute pancreatitis: a systematic review and meta-analysis of randomized controlled trials
Source: Crit Care. 2014 Mar 31;18(2):R57. doi: 10.1186/cc13809 (PMC4056604; doi:10.1186/cc13809)
Supplement: Additional file 1 — Systematic literature search for studies on the use of probiotics in critical illness. [file cc13809-S1.doc]

**Additional file 1. Systematic literature search for studies on the use of probiotics in critical illness**

A systematic literature search was performed in PubMed for RCTs from Jan 1^st^, 1992 to Jul 1^st^, 2013. The MeSH headings “critical illness,” “critical care,” “intensive care,” “intensive care units,” “lactobacillus,” “prebiotics,” “synbiotics” and “probiotics” were used. We excluded cohorts that did not report the outcome of infection. Finally, 13 RCTs were included for meta-analysis. Compared with a recent meta-analysis of the probiotics used in critical illness, which also included 13 RCTs, the only exception in our study was that we did not include an RCT that did not report infection , while the Giamarellos-Bourboulis 2009 RCT was included. The other studies are the same.

1. Rayes N, Hansen S, Seehofer D, Muller AR, Serke S, Bengmark S, Neuhaus P: **Early enteral supply of fiber and Lactobacilli versus conventional nutrition: a controlled trial in patients with major abdominal surgery**. *Nutrition* 2002, **18**(7-8):609-615.

2. Jain PK, McNaught CE, Anderson AD, MacFie J, Mitchell CJ: **Influence of synbiotic containing Lactobacillus acidophilus La5, Bifidobacterium lactis Bb 12, Streptococcus thermophilus, Lactobacillus bulgaricus and oligofructose on gut barrier function and sepsis in critically ill patients: a randomised controlled trial**. *Clinical nutrition* 2004, **23**(4):467-475.

3. McNaught CE, Woodcock NP, Anderson AD, MacFie J: **A prospective randomised trial of probiotics in critically ill patients**. *Clinical nutrition* 2005, **24**(2):211-219.

4. Rayes N, Seehofer D, Theruvath T, Schiller RA, Langrehr JM, Jonas S, Bengmark S, Neuhaus P: **Supply of pre- and probiotics reduces bacterial infection rates after liver transplantation--a randomized, double-blind trial**. *American journal of transplantation : official journal of the American Society of Transplantation and the American Society of Transplant Surgeons* 2005, **5**(1):125-130.

5. Kotzampassi K, Giamarellos-Bourboulis EJ, Voudouris A, Kazamias P, Eleftheriadis E: **Benefits of a synbiotic formula (Synbiotic 2000Forte) in critically Ill trauma patients: early results of a randomized controlled trial**. *World journal of surgery* 2006, **30**(10):1848-1855.

6. Spindler-Vesel A, Bengmark S, Vovk I, Cerovic O, Kompan L: **Synbiotics, prebiotics, glutamine, or peptide in early enteral nutrition: a randomized study in trauma patients**. *JPEN Journal of parenteral and enteral nutrition* 2007, **31**(2):119-126.

7. Klarin B, Molin G, Jeppsson B, Larsson A: **Use of the probiotic Lactobacillus plantarum 299 to reduce pathogenic bacteria in the oropharynx of intubated patients: a randomised controlled open pilot study**. *Critical care* 2008, **12**(6):R136.

8. Knight DJ, Gardiner D, Banks A, Snape SE, Weston VC, Bengmark S, Girling KJ: **Effect of synbiotic therapy on the incidence of ventilator associated pneumonia in critically ill patients: a randomised, double-blind, placebo-controlled trial**. *Intensive care medicine* 2009, **35**(5):854-861.

9. Morrow LE, Kollef MH, Casale TB: **Probiotic prophylaxis of ventilator-associated pneumonia: a blinded, randomized, controlled trial**. *American journal of respiratory and critical care medicine* 2010, **182**(8):1058-1064.

10. Barraud D, Blard C, Hein F, Marcon O, Cravoisy A, Nace L, Alla F, Bollaert PE, Gibot S: **Probiotics in the critically ill patient: a double blind, randomized, placebo-controlled trial**. *Intensive care medicine* 2010, **36**(9):1540-1547.

11. Oudhuis GJ, Bergmans DC, Dormans T, Zwaveling JH, Kessels A, Prins MH, Stobberingh EE, Verbon A: **Probiotics versus antibiotic decontamination of the digestive tract: infection and mortality**. *Intensive care medicine* 2011, **37**(1):110-117.

12. Tan M, Zhu JC, Du J, Zhang LM, Yin HH: **Effects of probiotics on serum levels of Th1/Th2 cytokine and clinical outcomes in severe traumatic brain-injured patients: a prospective randomized pilot study**. *Critical care* 2011, **15**(6):R290.

13. Giamarellos-Bourboulis EJ, Bengmark S, Kanellakopoulou K, Kotzampassi K: **Pro- and synbiotics to control inflammation and infection in patients with multiple injuries**. *The Journal of trauma* 2009, **67**(4):815-821.

14. Barraud D, Bollaert PE, Gibot S: **Impact of the administration of probiotics on mortality in critically ill adult patients: a meta-analysis of randomized controlled trials**. *Chest* 2013, **143**(3):646-655.

15. Alberda C, Gramlich L, Meddings J, Field C, McCargar L, Kutsogiannis D, Fedorak R, Madsen K: **Effects of probiotic therapy in critically ill patients: a randomized, double-blind, placebo-controlled trial**. *The American journal of clinical nutrition* 2007, **85**(3):816-823.
