# Supplementary material for: Use of probiotics in the treatment of severe acute pancreatitis: a systematic review and meta-analysis of randomized controlled trials
Source: Crit Care. 2014 Mar 31;18(2):R57. doi: 10.1186/cc13809 (PMC4056604; doi:10.1186/cc13809)

**Additional file 2.** **Risk of bias**

The figure indicates that five, four, four, four, one and five trials are at high or unclear risk of bias generated from random sequence generation, allocation concealment, blinding of participants and personnel, blinding of outcome assessment, incomplete outcome data, and selective reporting, respectively.


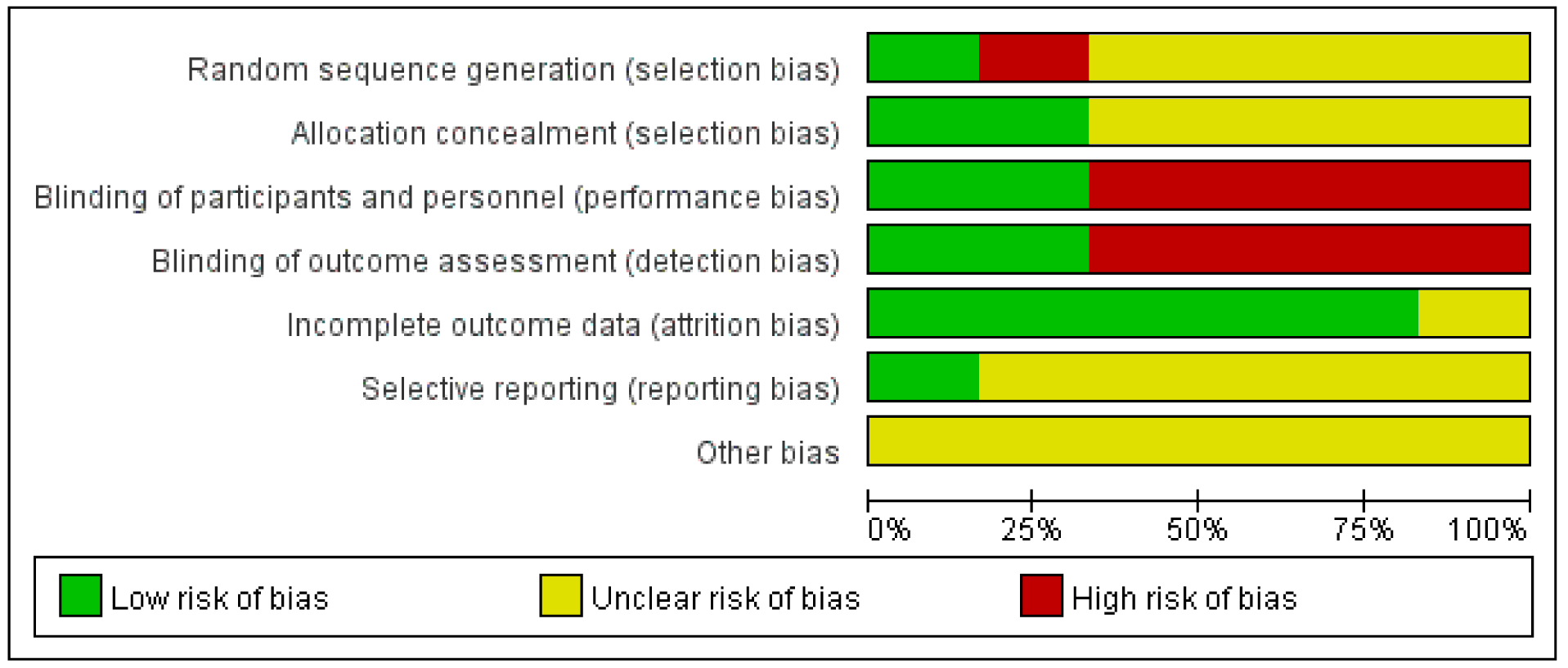

Supplement: Additional file 2 — Risk of bias. [file cc13809-S2.doc]
