# Supplementary material for: Use of probiotics in the treatment of severe acute pancreatitis: a systematic review and meta-analysis of randomized controlled trials
Source: Crit Care. 2014 Mar 31;18(2):R57. doi: 10.1186/cc13809 (PMC4056604; doi:10.1186/cc13809)
Supplement: Additional file 3 — Analysis of subgroups by treatment duration in predicted SAP. [file cc13809-S3.doc]

**Additional file 3.** **Analysis of subgroups by treatment duration in predicted SAP**

The forest plot illustrates the effects of probiotics administration on the clinical outcomes of patients at risk of severe acute pancreatitis. The heterogeneities within subgroups are small, but significant between subgroups, which suggest that treatment duration may be an important source of the heterogeneity. The results suggest that patients can benefit from probiotics when the treatment duration is within 15 days.
