# Supplementary material for: Use of probiotics in the treatment of severe acute pancreatitis: a systematic review and meta-analysis of randomized controlled trials
Source: Crit Care. 2014 Mar 31;18(2):R57. doi: 10.1186/cc13809 (PMC4056604; doi:10.1186/cc13809)
Supplement: Additional file 4 — Analysis of subgroups by types of probiotics used in critical illness. [file cc13809-S4.doc]

**Additional file 4.** **Analysis of subgroups by types of probiotics used in critical illness**

The forest plot illustrates the effects of probiotics administration on the clinical outcomes of patients with critical illness. The results suggest that there is heterogeneity among different types of probiotics. The *L. plantarum* 299, Synbiotic 2000, and Golden bifid have been also used for predicted SAP in one (Olah 2002), two (Olah 2007 and Plaudis 2012), and one (Li 2007) RCTs respectively. The probiotics or probiotic mixtures used in Jain 2004, Barraud 2010 and Morrow 2010 studies have not been used for predicted SAP in RCT.

*The probiotic mixture used in Jain 2004 study contained *L. acidophilus*, *B. lactis*, *S. thermophilus*, and *L. bulgaricus*. Two of the four probiotic strains (*L. acidophilus* and *B. lactis*) are also contained in Ecologic 641(*L. acidophilus*, *L. casei*, *L. salivarius*, *L. lactis*, *B. bifidum*, and *B. lactis*).

^#^ The probiotic mixture used in Barraud 2010 study contained *L. rhamnosus*, *L. casei*, *L. acidophilus*, and *B. bifidum*. Three of the four probiotic strains (*L. casei*, *L. acidophilus*, and *B. bifidum*) are also contained in Ecologic 641.

^§^The probiotics used in Morrow 2010 study was *L. rhamnosus*.

*

#

#

§

**References**

1. Olah A, Belagyi T, Issekutz A, Gamal ME, Bengmark S: **Randomized clinical trial of specific lactobacillus and fibre supplement to early enteral nutrition in patients with acute pancreatitis**. *The British journal of surgery* 2002, **89**(9):1103-1107.

2. Olah A, Belagyi T, Poto L, Romics L, Jr., Bengmark S: **Synbiotic control of inflammation and infection in severe acute pancreatitis: a prospective, randomized, double blind study**. *Hepato-gastroenterology* 2007, **54**(74):590-594.

3. Plaudis H, Pupelis G, Zeiza K, Boka V: **Early low volume oral synbiotic/prebiotic supplemented enteral stimulation of the gut in patients with severe acute pancreatitis: a prospective feasibility study**. *Acta chirurgica Belgica* 2012, **112**(2):131-138.

4. Li Y: **Adjuvant therapy for probiotics in patients with severe acute pancreatitis: An analysis of 14 cases**. *Shijie Huaren Xiaohua Zazhi* 2007, **15**(3):302-304.
