# Supplementary material for: Use of probiotics in the treatment of severe acute pancreatitis: a systematic review and meta-analysis of randomized controlled trials
Source: Crit Care. 2014 Mar 31;18(2):R57. doi: 10.1186/cc13809 (PMC4056604; doi:10.1186/cc13809)
Supplement: Additional file 5 — Analysis of subgroups by treatment duration in critical illness. [file cc13809-S5.doc]

**Additional file 5.** **Analysis of subgroups by treatment duration in critical illness**

The forest plot illustrates the effects of probiotics administration on the clinical outcomes of patients with critical illness. The results suggest that with regard to total infection and pneumonia, patients benefit more from probiotics when the treatment duration is within 15 days.
